# Family-integrated diabetes education for individuals with diabetes in South-west Nigeria

**DOI:** 10.4314/gmj.v56i4.6

**Published:** 2022-12

**Authors:** Lucia Y Ojewale, Oyeninhun A Oluwatosin

**Affiliations:** Department of Nursing, Faculty of Clinical Sciences, College of Medicine, University of Ibadan, Ibadan, Oyo State, Nigeria

**Keywords:** Family education, Diabetes-education, Glycosylated-haemoglobin, Diabetes-knowledge

## Abstract

**Objectives:**

This study aimed to determine the effects of family-integrated diabetes education on diabetes knowthe ledge of patients and family members, as well as its impact on patients' glycosylated haemoglobin (A1C).

**Design:**

The design was a two-group Pretest Posttest quasi-experimental.

**Setting:**

The study took place at the diabetes clinics of two tertiary hospitals in southwestern Nigeria.

**Participants:**

People Living with Diabetes (PLWD) and family members aged 18 years and over and without cognitive impairment were placed, as clusters, into either a control group (CG) or an intervention group (IG) The CG comprised 88 patients and 88 family members while IG comprised 82 patients and 82 family members. Of these, 78 and 74 patients completed the study in CG and IG, respectively.

**Interventions:**

PLWD in IG along with their family members were given an educational intervention on diabetes management and collaborative support with an information booklet provided. This was followed by three (3) complimentary Short Messaging Service (SMS).

**Main outcome measures:**

A1C and diabetes knowledge.

**Results:**

Over half (52.4%) and about a fifth (18.2%) of family members and patients, respectively, had never had diabetes education. There was a statistically significant increase in the knowledge of patients and family members in IG. Unlike CG, the A1C of patients in IG improved significantly at three and six-month post-intervention, (p<0.01). Regression showed an independent effect of family members' knowledge on IG's A1C.

**Conclusions:**

Improved family members' diabetes knowledge positively impacted patients' glucose level. There is a need to integrate family members into diabetes care better.

**Funding:**

African Doctoral Dissertation Research Fellowship (ADDRF) award offered by the Africa Population and Health Research Center (APHRC) in partnership with the International Development Research Centre.

## Introduction

Diabetes mellitus (DM) is a condition that affects not only the person living with diabetes (PLWD) but also the entire family.^1^ The knowledge and support of family members of PLWD are therefore central to effective diabetes care, particularly in many African countries, where family members relate in a close-knit manner. The greatest increase in the prevalence of diabetes is expected in Africa.^2^ Moreso, comparatively higher hospital admissions and case fatality rates have been reported in Nigeria.^3^ These are due to hyperglycaemic emergencies, diabetes-associated foot ulcers and cardiovascular diseases. Given the chronic nature of diabetes mellitus, PLWD have to make multiple daily decisions about managing their condition involving behavioural change^4^ Effective daily decisions lead to improvement in blood glucose levels and prevention of complications.^5^

Diabetes Self-Management Education (DSME) has been termed the ‘key’ to self -care amself-carend leads to successful health-related outcomes.^6^ It is enhanced when family members are involved, and social support is incorporated.^7^

In a survey among PLWD in Nigeria, Ojewale et al, 8 reported that perception of family support positively influenced diabetes self-management. Other authors had earlier stated that there is a positive correlation between perception of the family support and optimum glycaemic level, i.e. a reduction n glycosylated haemoglobin (A1C) level.^7^,^9^ This means that family members have to be well informed about diabetes to provide supportive care.

This has not been the case in many healthcare facilities in Nigeria, where family members of PLW type 2 Diabetes are yet to be well-integrated into diabetes care.^8^ Nevertheless, family members also experience diabetes burden, distress, and negative emotion, which is reduced when exposed to diabetes education, 10 thus making them more effective in providing support.

Studies on the effects of Family-Integrated (also called family-based or family-oriented) Diabetes Education (FIDE) on the knowledge of PLWD, the knowledge of family members and the A1C of PLWD have been reported. For instance, some authors 11,12 reported an improvement in the diabetes knowledge of PLWD following educational interventions that included family/social support. Similarly, family members were reported to show an improvement in diabetes knowledge following FIDE.^13^,^14^

Further, some interventional studies suggest that educational interventions involving family members have effectively reduced A1C through better medication and diet adherence.^14^–^16^. The interventions also led to an improvement in the diabetes knowledge of PLWD. However, another study 17 reported no evidence of improvement in A1C after FIDE.

The need to generate evidence to support policies on Family-Integrated Diabetes Education in Nigeria and the scarcity of Africa-based experimental or quasi-experimental studies in this area motivated the study. Hence, the broad aim of this study was to determine the effects of Family-Integrated Diabetes Education (FIDE) on A1C and diabetes knowledge of PLWD. The secondary objective was to determine the diabetes knowledge of family members of PLWD. Besides the health promotion model, the family system and social cognitive theories guided this study and have been published in a narrative review.^18^ More specifically, the study sought to answer the following research questions: what is the diabetes knowledge of PLWD before and after a family-integrated diabetes education in intervention and control groups?; what is the diabetes knowledge of family members of PLWD before and after a family-integrated diabetes education in intervention and control groups?; what is the glycosylated haemoglobin (A1C) level of PLWD in intervention and control groups at baseline and three and six-month post-intervention? And are there changes in the proportion of patients with sub-optimal A1C in the intervention group post-intervention?

## Methods

### Design, setting, participants and sampling

The study was a pre-test post-test quasi-experimental research involving two groups of PLWD – Type 2 and was part of a larger study between July 2016 and April 2017. An aspect of the study on ‘Patient Characteristics, Perception of Family Support and Diabetes Self-management has already been published.^8^ Cluster randomisation, in which all eligible participants in a particular hospital are selected, was used in allocating PLWD into intervention and control groups. This was done to prevent contamination in which intervention and control groups exchange information because of proximity. Hence, the two groups (intervention and control) consisted of registered PLWD in two purposively selected tertiary hospitals in South-western Nigeria. The two hospitals were selected because they had similar infrastructure and programmes for PLWD. These hospitals were University College Hospital (UCH) and Olabisi Onabanjo University Teaching Hospital (OOUTH). The two hospitals are about 80km from each other. A balloting system was used to determine which cluster would be the intervention and which would be the control group.

The study population consisted of PLWD - type-2 - aged ≥18years with an adult family member living in the same household, who consented to accompany the patient and participate in the study. Other eligibility criteria for the PLWD included the absence of cognitive impairment, pregnancy and/or diabetes complication (s) such as nephropathy. Eligibility criteria for family members included being at least 18 years, not having been diagnosed with diabetes and not having cognitive impairment. A purposive sampling technique was used in selecting participants who met the inclusion criteria because not all the PLWD could get their family members to participate in the study. Using the sample size formula for determining differences in proportion between two independent groups; the prevalence of sub-optimal A1C among PLWD in Nigeria of 64%, 19, an estimated effect size of 25% and a power of 85%; a total of 170 PLWD – 88 in intervention and 82 in the control group with a corresponding number of family members were recruited at baseline.

### Instrument

A questionnaire comprised of two parts was used to elicit information from PLWD. The first section consisted of socio-demographic and clinical-related information based on a literature search on related studies. The second section of the questionnaire contained a modified and adapted form of the 14-item Diabetes Knowledge Test (DKT). The DKT was developed by the Michigan Diabetes Research Training Centre (MDRTC) in 1998.^20^ The DKT-14 consisted of 14 general questions on diabetes, and the questions were in multiple-choice format, with only one correct answer.

The Cronbach's alpha of the DKT was 0.83 after its modification and adaptation, using PLWD in another health facility different from the ones used for the study.

Family members also completed a questionnaire consisting of an adapted version of the DKT, in addition to providing relevant socio-demographic data. The reliability score for the family members' version of DKT during the pilot study was 0.91. The pilot study took place at Ladoke Akintola University Teaching Hospital, (LAUTECH), Ogbomoso town in Oyo State, Nigeria.

An A1C Point of Care (POC) analyser (A1c Now+), which was manufactured by Polymer technology (USA), was used in checking the A1C of the PLWD in both study facilities. The researcher carried out the A1C check out while trained research assistants helped in administering the questionnaire to the PLWD and their family members. A teaching module was adapted for diabetes patients and their family members based on the IDF diabetes education curriculum for sub-Saharan Africa.^21^ The module, in addition to highlighting key areas of diabetes education such as management, prevention of complications, also emphasises the psychosocial impact of diabetes including the role of family members. The module was made into a booklet.

### Ethical clearance and data collection.

Ethical approvals were granted by the University College Hospital/University of Ibadan Institutional Review Board (IRB) and Olabisi Onabanjo University Teaching Hospital Health Research Ethics Committee (HREC). The approval numbers were UI/EC/15/0012 (UCH) and OOUTH/HREC/031/2015 (OOUTTH). The permission of the Head of the Department of Endocrinology units and the collaboration of nurse educators was sought and obtained. Informed consent was sought and obtained in written form from each study participant. Participants' recruitment and participation were in line with the ethical principles of beneficence, confidentiality, autonomy and nonmaleficence. All participants, i.e., PLWD and family members, were given transportation fare after data collection

### Pre-intervention (Phase 1 – P1)

An initial visit was made to both UCH and OOUTH, which were the control and intervention hospitals respectively after the ethical approval had been obtained. During these visits, the nature of the study, the purpose, its requirements (eligibility criteria) as well as the benefits were explained to the PLWD. The nurses and physicians were carried along during this planning phase. A purposive sampling of all eligible patients who gave consent to participate in the study was done. Participant recruitment lasted for three months.

A questionnaire on diabetes knowledge was administered to the patients and family members, followed by an A1C check. Participants in the intervention group were told about the date and venue of the diabetes education.

### Intervention (Phase 2 - P2)

The Family-Integrated Diabetes Educational (FIDE) programme for participants in the intervention group (comprising three batches) lasted for one day – approximately five (5) hours each day. This face-to-face educational programme was complemented by three educational Short Message Services (SMS) sent to family members, due to the inability of family members to obtain more than one-day permission from their workplaces. The educational programme took the form of lecture/ discussion and goal setting among family member units. The family units were divided into four groups comprising an average of 20 family units. One group was taken on the Wednesday of each week, hence the intervention lasted for four (4) weeks. The overall theme of the education intervention was ‘Patient-Family Collaboration in Diabetes Care’. There were six sections in the module namely: Introduction and overview of diabetes; dietary management; blood glucose target including self - glucose monitoring and signs and symptoms of hypoglycaemia/ hyperglycaemia; physical activities/ exercise; use of tablets or insulin and family collaboration in management. Each patient and family member received the booklet. The educational intervention was given by the Researcher who spoke the native and English languages, fluently. Research assistants helped in setting up audiovisual aids and in managing the participants. The seminar was followed by three (3) monthly SMS messages to family members reminding them of concrete ways in which they could assist/support the patient in managing diabetes. All participants were provided with transportation fare and refreshments.

### Post Intervention Phase (Phases 3 & 4 – P3 & P4)

Immediately after the intervention, the questionnaire sections on the knowledge-diabetes knowledge test (DKT) were administered to the PLWD and family members separately. The same procedure was followed for the participants in the control group, except that they did not have the intervention.

During the three and six-month post-intervention follow-up, the A1C of PLWD was checked for both intervention and control groups. The PLWD were provided with transportation fare during the follow-up visit as well. Furthermore, for ethical reasons, the same educational intervention given to participants in the intervention group was repeated for those in the control group at the end of the study. PLWD and family members who attended this seminar also received the booklet printed for the study and were provided with refreshments.

### Data analysis

Questionnaires were checked daily to ascertain whether they were properly completed and for errors. Data were then entered into a computer software - IBM – SPSS version 22 for analysis. Participants' socio-demographic distribution across study groups was analysed using frequencies and percentages while Chi-square and independent t-tests were used in comparing baseline characteristics of the two study groups. The maximum obtainable score on DKT was 14, while the lowest was zero (0). One (1) mark was allocated to each correct answer, while none was allocated if a question was missed. The mean score on DKT was determined for both PLWD and family members. Between-group analysis was done using an independent t-test. Simple linear regression was used in determining the independent effect of family members' knowledge on A1C. Mean A1C values were calculated, and these values were further categorised into ‘below target’ (> 7%/ > 53mmol/mol) and target A1C (≤ 7%/ ≤ 53mmol/mol) for both intervention and control groups. The authors used Repeated measures ANOVA to com-pare the A1C measures at baseline, three-month and six-month post-intervention.

## Results

One hundred and seventy (170) PLWD - type 2 with the same number of family members were recruited into the study. Out of the 170, 88 were recruited as the Control Group (CG) from the UCH and 82 as Intervention Group (IG) from OOUTH. The 170 pairs of participants took part in Phase 2 of the study. Out of these, a total of six (6) dropped out by the time of the 3-month post-intervention follow-up (P3) - three (3) from each of the two groups. Thus, a total of 164 patients completed the P3. The six-month follow up (P4) involved a total of 152 patients with 78 from IG and 74 from CG. This was because twelve patients had dropped out - seven (7) from CG and five (5) from IG.

### Socio-demographic and clinical data of PLWD and family members

The socio-demographic and clinical variables of the PLWD are presented in Table 1. Most study participants were female (70.0%), with 55.9% aged 60 years and above. About a third (35%) were on insulin therapy, while 75.4% had been exposed to diabetes education. Majority (80.6%) of the PLWD had a personal glucometer. There was no statistical difference in the socio-demographic data of the two groups. Family members' characteristics and comparisons of these are presented in Table 2. They were predominantly female (63.6%) and a little over half (53.4%) were aged 40 years and below.

**Table 1:** Comparison of the socio-demographic characteristics of PLWD in intervention and control groups using chi-square test

Variable	Control group	Intervention group(n=82)		
	(n=88)	Frequency (%)	Total (%)	p- value
	Frequency (%)			
**Sex: Male**	23 (26.1)	28 (34.1)	51 (30.0)	0.315
**Female**	65 (73.9)	54 (65.9)	119 (70.0)	
**Marital status**				
**Married**	63 (71.6)	62 (75.6)	125 (73.5)	0.604
**Not married**	25 (28.4)	20 (24.4)	45 (26.5)	
**Age: ≤40 years**	4 (4.0)	6 (6.1)	10 (5.9)	
**41–59 years**	28 (31.8)	27 (45.1)	65 (38.2)	0.106
**≥60 years**	56 (63.6)	39 (47.6)	95 (55.9)	
**Minimum**	31	27		
**Maximum**	83	80		
**Use of insulin injection:**				
**Yes**	30 (34.1)	29 (35.4)	59 (35)	0.873
**No**	58 (65.9)	53 (64.6)	118 (65)	
**Educational level:**				
**Tertiary**	29 (33.0)	27(32.9)	56 (33)	1
**≤ Secondary**	59 (67.0)	55 (67.1)	114 (67)	
**Ownership of a** **glucometer:** **Yes**	76 (86.4)	61 (74.4)	137 (80.6)	0.054
**No**	12 (13.6)	21 (25.6)	33 (19.4)	
**Previous DM** **education: Yes**	75(85.2)	64 (78.0)	139 (81.8)	0.24
**No**	13 (14.8)	18 (22.0)	31 (18.2)	
**Diabetes duration:**				
**< 20 years**	76 (86.4)	72 (88.9)	148 (87.6)	0.649
**≥ 20 years**	12 (13.6)	9 (11.1)	21 (12.4)	
**Family member:** **Spouse**	22 (25.0)	28 (34.1)	50(29.4)	0.363
**Child**	51 (58.0)	44 (53.7)	95 (55.9)	
**Others**	15 (17.0)	10 (12.2)	25 (14.7)	

There was no significant difference in the characteristics of PLWD in the intervention and control groups at baseline.

**Table 2:** Baseline characteristics and comparison of family members across study groups using chi-square test

Variable	Control group	Intervention group		
	f (%) (n = 88)	f (%) (n=82)	Total (%)	p - value
**Sex: Male**	32 (36.4	31 (37.8)	63 (37.1)	0.875
**Female**	56 (63.6)	51 (62.2)	107 (62.9)	
**Age (in years)**				
**≤ 40 years**	47 (53.4)	41 (50.0)	88 (51.8)	0.759
**> 40 years**	41 (46.6)	41 (50.0)	82 (48.2)	
**Mean (SD)**	41.7 (16.7	40.0 (15.1)		
**Educational level:**				
**Tertiary:**	52 (59.1)	37 (45.1)	89 (52.4)	0.091
**Secondary & Below**	36 (40.9)	45 (54.9)	81 (47.6)	
**Previous DM education**				
**Yes:**	40 (45.5)	41 (50)	81 (47.6)	0.645
**No:**	48 (54.5)	41 (50)	89 (52.4)	
**Self-rating of DM knowledge:**				
**Good:**	18 (20.5)	21 (25.6)	39 (22.9)	0.603
**Average:**	42 (47.7)	40 (48.8)	82 (48.2)	
**Poor:**	18 (20.5)	16 (19.5)	34 (20.0)	
**Non - existent:**	10 (11.4)	5 (6.1)	15 (8.8)	
**Previous DM education**				
**Yes:**	40 (45.5)	41 (50)	81 (47.6)	
**No:**	48 (54.5)	41 (50)	89 (52.4)	

The family members of PLWD were not significantly different in their characteristics at baseline.

The Chi-square test showed no significant difference in the characteristics of the family members.

### Diabetes knowledge of PLWD and family members pre- and post-intervention

The comparison of the mean diabetes knowledge score of PLWD and family members in the intervention and control groups is presented in Table 3. It shows that there was no significant difference in the scores at baseline. However, post-intervention, PLWD and their family members in the intervention group displayed a significantly higher score with a p-value < 0.01.

**Table 3:** Comparison of diabetes knowledge (DKT) of PLWD and family members between groups at pre- and post-intervention using Independent t-test

		Control		Intervention				
Group	Study Phase	n	*x̄* (±)	n	*x̄* (±)	mean diff.	t- value	p-value
**PLWD**	**P1**	88	6.1 (2.3)	82	5.8 (2.4)	0.334	0.932	0.352
	**P2**	88	6.1 (2.3)	82	9.7 (2.6)	- 3.559	- 9.505	<0.01**
**Family** **members**	**P1**	88	5.9 (2.3)	82	5.6 (2.4)	- 0.448	1.343	0.181
	**P2**	88	5.8 (2.2)	82	8.6 (3.0)	2.84	-7.1	<0.01*

P1: Pre-intervention P2: Post-intervention

** Significant at p < 0.01

PLWD in the intervention group had significantly higher diabetes knowledge after the educational intervention (P2). Similarly, family members in the intervention group scored significantly higher on diabetes knowledge post-intervention.

The result of the simple linear regression analysis, presented in Table 5, regarding the effect of family members' knowledge on A1C independently and when PLWD's knowledge was adjusted for was statistically significant.

**Table 5:** Independent t-test on comparison of glycosylated haemoglobin (A1C) of participants during the three phases of the study

Study phases	Study group	Mean (SD.)	mean diff.	95% CI UCI, LCI	t – value	p value
**P1**	Control	7.5 (2.1)	-1.2	-1.815; -0.585	-3.854	< 0.01*
	Intervention	8.6 (2.2)				
**P3**	Control	8.0 (2.1)	0.314	-0.253; 0.881	1.094	0.276
	Intervention	7.7 (1.5)				
**P4**	Control	7.8 (2.1)	0.302	-0.380; 0.849	0.938	0.35
	Intervention	7.5 (1.8)				

P1: Pre-intervention P3: three-month post-intervention P4: six-month post-intervention

UCI: Upper Confidence Interval, LCI: Lower Confidence Interval

* Significant at p < 0.01

### Glycosylated haemoglobin (A1C) values pre- and post-intervention

Independent t-test at baseline, presented in Table 6, shows a significant difference in the A1C of the two groups but no significant difference in the two groups at post-intervention. However, the repeated measures ANOVA as shown in table 7 indicates that PLWD in the intervention group had a significant reduction in the A1C level at three and six- months post-intervention compared to the baseline value.

**Table 6:** Repeated measures ANOVA showing the A1C level of PLWD in the intervention and control groups during the three study phases

Study group Phase		Mean	mean diff.	95% CI (UPI; LCI)	p – value
**Intervention**	P1	8.9	-1.123	.616; 1.630	<0. 01**
	P3	7.8			
	P1	8.9	-1.341	.738; 1.943	<0. 01**
	P4	7.5			
	P3	7.8	-0.218	-.218; .654	0.225
	P4	7.5			
**Control**	P1	7.4	0.694	-1.140; -.247	<0.01**+
	P3	8			
	P1	7.4			
	P4	7.8	0.487	-.933; -.041	0.009*
	P3	8	-0.206	-.202; .615	0.22
	P4	7.8			

P1: baseline, P3: three-month post-intervention; P4: six-month post-intervention

** significant at < 0.01

+ change is negative

**Table 7:** Proportional changes in A1C level among intervention and control groups during the three phases of the study and comparison using a chi-square test

		Control			Intervention		
A1C level	normal	high	Total	normal	high	Total	
Study phase	req. (%)	freq. (%)	freq. (%)	freq. (%)	freq. (%)	freq. (%)	p-value
**P1**	43 (48.9)	45 (51.1)	88 (100)	27 (32.9)	55 (67.1)	82(100)	0.012*
**P3**	33 (38.8)	52 (61.2)	85 (100)	35 (44.3)	44 (55.7)	79 (100)	0.29
**P4**	30 (38.5)	48 (61.5)	78 (100)	34 (45.9)	40 (54.1)	74 (100)	0.221

* There was a statistically significant difference at baseline. PLWD in the Control group ad A1C close to the target, i.e., normal.

PLWD in the control group had a significantly lower mean A1C value at baseline. However, there was no significant difference in the two groups' mean A1C at three and six-month post-intervention. There was a significant reduction in the A1C of PLWD in the intervention group at three and six-month post-intervention. On the other hand, there was a significant increase in the control group signifying a worsening of glycaemic control.

Finally, the change in the proportion of PLWD who were able to achieve the target A1C of ≤ 7% is presented in Table 8. The proportion of PLWD in the intervention group with target A1C increased by 13%, whereas the CG had a higher proportion not meeting the target A1C.

There was a 13% increase in the proportion of PLWD having a target glucose value in the intervention group at six-month post-intervention. Those in the control group did not maintain the target A1C level.

## Discussion

People Living with Diabetes (PLWD) in the intervention and control groups showed less than average knowledge. of DM at baseline, with both groups having mean scores less than half of the maximum score.

This agrees with Adejoh et al., 15, who reported that nearly half of PLWD in a Nigerian hospital exhibited little diabetes knowledge. The difference in the post-intervention knowledge score was significantly higher among PLWD in the intervention group compared to the control group.

This is in line with the findings of Ahmed et al, 22, who reported a significant increase in the post-intervention knowledge of PLWD in Egypt after an educational intervention. Similar findings were reported by Wichit et al., 17 and Hu et al 13 after a family-oriented diabetes intervention programme. Baig et al., 23 further reported significant improvement in the knowledge of elderly PLWD following spousal involvement in DSME.

In a review of 26 family-based interventions for adults with type 2 DM, Baig et al 23 stated that very few studies measure the family outcome. Also, Kovacs et al, 10 in a multi-national study involving seventeen countries in four continents, (including Africa), on Diabetes Attitude, Wish and Needs (DAWN) of family members of people with diabetes revealed that only 23% of family members had ever participated in diabetes education. Out of these, 72.1% stated that education helped them understand diabetes and offer emotional support to their sick relatives. This study assessed family members' knowledge at baseline and immediately after the intervention. Despite baseline comparability, the family members in the intervention group showed a significant improvement in knowledge compared to the control group at post-intervention. This finding is in line with that of Hu et al., 14 among Hispanics in North Carolina as well as that carried out among Chinese PLWD and their family members.^13^ Moreover, the mean A1C value of PLWD in the intervention and control groups at baseline, 8.9% and 7.4%, respectively, are relatively similar to the 8% reported by Adebisi et al., 19 among PLWD in a Nigerian hospital. In a later study that cut across seven tertiary hospitals in the six geopolitical zones of Nigeria, authors reported a mean value of 8.3%. 24 Further, Oghagbon, 25 reported a range of 7.9% to 8.3% among PLWD managed over 12 years in one Nigerian hospital. These high values show that more interventions need to be carried out regarding the glycaemic target of PLWD since the normal/recommended A1C value is 7%.^26^ Participants in this study's intervention group achieved some improvement in glycaemic level.

PLWD in the intervention group had a significantly higher level of A1C than their counterparts in the control group at the study commencement. This study's main limitation and could not be forestalled due to the use of cluster randomisation in participant selection. Nevertheless, the within-group analysis shows that PLWD in the intervention group had a significant reduction in their A1C level at three months post-intervention, signifying improvement in glycaemic level; the PLWD in the control group had a significant increase in A1C level at the three-month follow-up.

The worsening of the A1C target among the control group, compared to the baseline value could be associated with binge eating during the festive period, as suggested by some of the PLWD in the group.

The improvement in glycaemic level in the intervention group is in keeping with the findings of Hu et al. 27 who carried out a one-group pre-test post-test intervention study. The finding is also similar to that of García et al.^28^ Moreover, the PLWD in the intervention group still had a reduction in the glycaemic level at six-month post-intervention. This finding agrees with that of Pamungkas et al.^7^ Even though the A1C level of PLWD in the intervention group decreased significantly following the intervention, the baseline incompatibility made it difficult to have a significant difference between the two groups.

Furthermore, in this study, the A1C level of PLWD in the intervention group decreased by 1.1%, at three – month post-intervention. This level of improvement is similar to that reported by other authors, as stated by Pillay et al, 29 in a systematic review and network meta-analysis for effect moderation on behavioural programmes for type 2 DM. The author further observed that Diabetes Self-Management Education (DSME), that included support programmes with a duration ≥ 11 contact hours, caused a minimum of 0.4% reduction in A1C. Based on the results of this study, it could be asserted that the educational intervention, though less than 11 hours, but which was followed by SMS text messages and the provision of a diabetes education booklet, was effective in the achievement of a substantial reduction in A1C level.

Likewise, at six-month post-intervention, the A1C level of PLWD in the intervention group had decreased by 1.4%. As little as one per cent (1%) decrease in A1C is reported to be associated with a 21% reduction in diabetes-related mortality, 37% reduction in the risk of developing microvascular complications, and a 14% reduction in the risk of developing myocardial infarction.^30^

A review of the method and structure of diabetes education to include family members actively is necessary for diabetes clinics in South-west Nigeria. This will ensure better assimilation for the PLWD which will translate to better self-care. Periodic evaluation of the diabetes knowledge of PLWD and their family members could help nurses, and diabetes care and education specialists identify areas of knowledge deficiencies. Although not reported in this study, the family-integrated educational intervention promoted open discussion among family members and would have impacted the quality of life of PLWD. This is another reason why this practice should be made habitual.

Study limitations include restricting the study to only PLWD whom a family member could accompany. This might have caused a selection bias. In addition, the intervention could have been longer but family members who were mostly in the working class could only obtain a day's permission from work. Finally, there were only two clusters, which limited the degree of freedom for calculating intra-cluster correlation (ICC)

## Conclusion

This paper highlighted the effects of family-integrated diabetes education on diabetes knowledge and glycosylated haemoglobin of PLWD - type 2 diabetes in South-western Nigeria. PLWD in both intervention and control groups demonstrated a less-than-average knowledge of diabetes mellitus at the baseline, despite many of them having attended diabetes education in the past. It appeared that the family-integrated diabetes education, complemented by SMS messages and the provision of an educational booklet for both PLWD and their family members in the intervention group, led to a better understanding of diabetes in both PLWD and their family members. The control and intervention groups had significantly different A1C levels, albeit high, at baseline due to cluster randomisation. PLWD in the intervention group achieved a significant improvement in their A1C level at three and six-month post-intervention. The implications for clinical practice were highlighted.

## Figures and Tables

**Table 4:** Simple linear regression analysis showing the independent effect of family members' knowledge on A1C while holding the knowledge of PLWD constant

	β	P-value	Lower CI	Upper CI
**Independent** **Knowledge of** **family members**	0.089	0.033	-.0.172	-.007
**Knowledge of** **family members** **adjusting for** **Knowledge of** **PLWD**	0.096	0.024*	-0.179	-0.013
**Knowledge of** **family members** **adjusting for** **Hba1c**	-0.107	0.086	-0.230	0.015

* Significant at p < 0.05

C.I: Confidence interval at 95%. Family members' knowledge independently improved the A1C of PLWD, while adjusting for knowledge of PLWD.
